# The role of substance use and morality in violent crime - a qualitative study among imprisoned individuals in opioid maintenance treatment

**DOI:** 10.1186/1477-7517-11-24

**Published:** 2014-08-20

**Authors:** Ingrid Amalia Havnes, Thomas Clausen, Christina Brux, Anne-Lise Middelthon

**Affiliations:** 1 Division of Mental Health and Addiction, Oslo University Hospital, PO Box 4956, Oslo, Nydalen 0424, Norway; 2 SERAF - Norwegian Centre for Addiction Research, University of Oslo, PO Box 1039, Oslo, Blindern 0315, Norway; 3 Institute of Health and Society, University of Oslo, PO Box 1130, Oslo, Blindern 0318, Norway

**Keywords:** Violence, Benzodiazepines, Opioid maintenance treatment, Morality, Prison, Crime, Qualitative methods, Antisocial personality disorder

## Abstract

**Background:**

Opioid maintenance treatment (OMT) is regarded as a crime control measure. Yet, some individuals are charged with violent criminal offenses while enrolled in OMT. This article aims to generate nuanced knowledge about violent crime among a group of imprisoned, OMT-enrolled individuals by exploring their understandings of the role of substances in violent crime prior to and during OMT, moral values related to violent crime, and post-crime processing of their moral transgressions.

**Methods:**

Twenty-eight semi-structured interviews were undertaken among 12 OMT-enrolled prisoners. The interviews were audio recorded and transcribed verbatim. An exploratory, thematic analysis was carried out with a reflexive and interactive approach.

**Findings:**

Prior to OMT, substances and, in particular, high-dose benzodiazepines were deliberately used to induce ‘antisocial selves’ capable of transgressing individual moral codes and performing non-violent and violent criminal acts, mainly to support costly heroin use. During OMT, impulsive and uncontrolled substance use just prior to the violent acts that the participants were imprisoned for was reported. Yet, to conduct a (violent) criminal act does not necessarily imply that one is without moral principles. The study participants maintain moral standards, engage in complex moral negotiations, and struggle to reconcile their moral transgressions. Benzodiazepines were also used to reduce memories of and alleviate the guilt associated with having committed violent crimes.

**Conclusions:**

Substances are used to transgress moral codes prior to committing and to neutralize the shame and guilt experienced after having committed violent crimes. Being simultaneously enrolled in OMT and imprisoned for a (violent) crime might evoke feelings of ‘double’ shame and guilt for both the criminal behavior prior to treatment and the actual case(s) one is imprisoned for while in OMT. Treatment providers should identify individuals with histories of violent behavior and, together with them, explore concrete episodes of violence and their emotional reactions. Particular attention should be given to potential relationships between substance use and violence and treatment approaches tailored accordingly. What appears as severe antisocial personality disorder may be partly explained by substance use.

## Background

Violent behavior cannot be attributed to any single factor; rather, a complexity of individual and environmental factors is involved [1-3]. Previous research identifies the following as among important factors associated with increased violence risk: earlier violent crime [4], ‘history of problems with other antisocial behavior’ [3], severe personality disorders [5-7], and substance abuse [8,9]. Most psychoactive substance use occurs among individuals who do not behave violently [8]. Nonetheless, the relationship between psychoactive substances and violence warrants attention and can be conceptualized as a tripartite model [10,11]: the *systemic* violence involved in the illegal drug market [10,12-14], the *economic compulsive* violence enacted to support costly drug use, and heroin use in particular [15], and the *psychopharmacological violence* that can occur during substance use, including acute intoxication, drug-seeking behavior associated with withdrawal, and episodes of drug-induced psychosis and paranoid symptoms [8,9].

Research suggests that alcohol is the substance most closely associated with aggression and violent behavior [8,9,16]. Violence has also been associated with the influence of stimulants like amphetamines/methamphetamines [17,18] and cocaine/crack cocaine [19,20]. Heavy and frequent use of amphetamines is also related to violence [17]. Disinhibitory benzodiazepine reactions, such as hostility, agitation, and loss of impulse control, have been reported [21,22], and high-dose benzodiazepine use is found to be a high risk factor for interpersonal violent crime [23]. Opioid use depresses activity and, thereby, temporarily inhibits violent behavior. During withdrawal, physical discomfort and agitation can result in violent behavior [8].

Opioid dependence is often associated with criminal activity [24-26]. Reductions in acquisitive and drug-related crime during opioid maintenance treatment (OMT) are widely reported [27-29]. Violent crime is found to be relatively uncommon among heroin users [30-32] and to decrease during OMT [33,34]. However, one group of individuals in OMT who had been convicted of violent crime prior to enrollment was found to have a higher risk of both violent and non-violent criminal convictions during OMT [34].

There is a need to gain a deeper understanding of criminal behavior among persons enrolled in OMT and, in particular, among those who exhibit violent behavior during treatment, thereby enabling treatment providers to tailor their treatment approaches accordingly and strategically incorporate violence risk management. This article aims to generate multifaceted and nuanced knowledge about violent crime among a group of imprisoned, OMT-enrolled individuals by exploring their understandings and articulations of the role of substances in violent crime prior to and during OMT, moral values related to violent crime, and post-crime processing of their moral transgressions.

## Methods

The exploratory, qualitative study upon which this and two other articles [35,36] are based formed part of a larger study focusing on criminal convictions in a national OMT cohort [29,34]. Violence, in this study, is defined as actual, attempted, or threatened bodily harm to another person [3].

### Setting

The Norwegian OMT program started in 1998 and was intended to reach a population of severely dependent heroin users who were not benefiting from other types of treatment [37]. Imprisoned, opioid-dependent individuals, including those serving long sentences, may enter the national OMT program. For individuals who are already enrolled in OMT when imprisoned, the treatment is continued [38].

### Sampling and recruitment

The inclusion criteria were that the study participants were of or above legal age, in a state to provide informed consent and enrolled in OMT at the time of imprisonment for suspected crime. To minimize recall bias, the study design set a short time span between the arrest and the first interview in prison, thereby targeting remand prisoners. Recruitment occurred in prisons; prison staff contacted the prisoners and those who agreed to meet the first author were given written and verbal information about the study. Three individuals chose not to participate, explaining that they needed time to prepare for court or did not want to discuss negative life experiences or sensitive matters.

### Data collection and analysis

28 one-hour long interviews were carried out in prison with the 12 participants. The interviews were conducted by IAH, who is a trained specialist in psychiatry. Most of the participants were interviewed between two and four times, though two individuals were interviewed only once due to short notice release from the remand wing. Given the exploratory character of the study, the interview guide was regularly revised [39,40]. We sought to narrativize topics [41] in order to facilitate nuanced, detailed, and concrete accounts of participant experiences, from their own perspectives.

To access normative responses on sensitive subjects, we sometimes utilized vignettes [42] and individualized guides were developed for subsequent interviews to validate findings from previous interviews [39] and facilitate cross-case analysis. Among the topics explored were the following: experiences with OMT, understandings of crime and violent behavior, understandings of substance use and its role in violent crime prior to and during OMT, norm systems and moral codes, life situations before imprisonment, and mental and physical health. The interviews were audio recorded, with the exception of one individual who preferred note-taking. Identification and analysis of emerging themes was jointly carried out by IAH and ALM as a ceaseless task that was integrated throughout the entire research process [43], and CB took part in the final stages of thematic analysis.

The biosocial model of violence and antisocial behavior [2] suggests that genes, environment, social, and biological factors can predispose individuals to aggression and violence and that biological and social risk factors can be linked to dysregulation of cognitive (thinking), affective (emotions), and motor (behaviors) brain processes. The findings from the thematic analysis have a special focus on how substance use were experienced by the participants to affect thinking, emotions, and behavior related to violent crime.

### Sample characteristics

Twelve persons, nine men, and three women between 22 and 50 years of age, participated in the study. Ten individuals were formerly convicted of several accounts of violent crime. Four of the individuals had been released from prison between 1 week and 1 month prior to being reinstated in prison when they were included in this study. There were eight cases of imprisonment for violent offenses during the study period. Though we are obliged to remain vague for the sake of upholding ethical standards, the types of violence they were imprisoned for during OMT vary, including violent threats and physical violence towards family members and staff in the treatment system, threats and physical violence against police officers during arrest, grievous robberies, severe interpersonal violence, and interpersonal violence resulting in death.

Time previously served in prison ranged from 1.5 to 20 years. All participants had been opioid dependent and poly-drug users for nearly 10 years or more prior to OMT. Half the sample was homeless and, at the time of our first interview, time spent in OMT varied from a few months to nearly 10 years. All participants had tight medication control regimes due to non-compliance with treatment regulations at the time of imprisonment [36].

### Ethics

The study was approved by the Regional Committee for Medical Research Ethics, the Norwegian Social Science Data Services and the Norwegian Correctional Service Region East. All participants provided voluntary and written informed consent. In addition to the formal requirements, emphasis was placed on ensuring anonymity throughout the publication process.

## Findings

This exploratory study generated rich, empirical material on a number of phenomena related to criminal and, in particular, violent behavior among OMT-enrolled individuals prior to imprisonment. In this article, we focus on the participants' *experiences and understandings of substance use and altered perceptions of reality and behavior related to violent behavior*, mostly prior to OMT, and *experiences of uncontrolled substance use and violence during OMT*. The last part of this section explores *moral principles related to violent behavior*, and particular attention is given to how the participants understand, rationalize, and/or struggle to reconcile their violent behavior.

### Drug use and altered perceptions of reality and behavior prior to OMT

The participants of this study commonly recognized the ways in which various substances influence their behavior and contribute to violent crime. While many reported having induced violent and criminal behavior through instrumental drug use prior to OMT, they explained that, while enrolled in OMT, their substance use, though still contributing to violent behavior, is most often impulsive. Victor had been a drug dealer and debt collector for several years prior to enrolling in OMT. He has served nearly a decade in prison for property crimes, drug-related crimes, robberies, and interpersonal violence. He is well aware of his ability to temporarily alter his ‘personality’ by taking pills and, in an effort to enable criminal behavior, deliberately sought certain changes prior to OMT. He continues to do so while enrolled in OMT, but to a lesser extent:

I could eat 20–30 pills [flunitrazepam and clonazepam]. [..] – Then you change… poor contact with others and reality. Even though I'm well aware of how I change, I do it sometimes every now and then, that's why I get imprisoned, like now… When I take these pills, I change personality completely. I am not a nice person then. I get mean. My reality… I become unconcerned. I don't give a damn about what people say. If you try to stop me, don't wear a uniform or happen to be two persons… then you have to watch out, because you restrict me in my world - inside my head. Because inside my head this is normal. And when I come home, I sit down to watch TV and start to think: ‘shit, what have I done now?’ Then it's too late, and you might use pills the next day or later because you'd rather forget about it.

Victor participates in an anger management program and recognizes that he has a problem with violent behavior in general and, particularly, when his perception of reality is altered due to the influence of high-dose flunitrazepam. Based on his description above, he could then be temporarily characterized as callous, unempathetic, and hostile; but, after committing a violent crime, he is compelled to use more pills to reduce guilt, alleviate remorse, and attempt to forget.

Impulsivity is a personality trait that several of the participants claim to have, and one that several experience to be enhanced while using substances and, particularly, high-dose benzodiazepines.

Simon has a long history as a drug dealer prior to OMT and provides yet another example of altered behavior while under the influence of drugs. He had, at the time of the interview, served several sentences for convictions of violence. Prior to OMT, Simon frequently used flunitrazepam instrumentally to decrease inhibitions and enable himself to commit crimes. During OMT, this happened on a few occasions:

Pills get you damn impulsive… I've usually taken pills if I commit crimes. I don't have the nerve to do it when I'm sober.

He claimed that his criminal behavior was greatly reduced following OMT enrollment. However, when collecting his daily methadone dose from the pharmacy, he was accessible to illicit drug dealers who offered him ‘pills’ on a daily basis. He explained that, on a bad day, he might accept, even though he knew that this impulsive flunitrazepam use may subsequently lead to violence and crime. He defines a ‘relapse’ as taking 10 mg or more of flunitrazepam and, when asked how this drug affects him, responds:

I get damn aggressive. Rude. When it comes to crime and such, I overcome barriers, lose inhibitions… I've always been high [on flunitrazepam and alcohol], when I've been convicted of violence.

Finally, Simon was also clear that regular use of benzodiazepines makes him more aggressive. But, as he saw it, the manner and dose in which benzodiazepines are used is of importance for instrumentally reducing inhibitions and/or inducing impulsivity and aggressive behavior:

One valium [diazepam] doesn't get you high. It's the way you eat it. There's a difference between shaving your head and cutting it off.

Several participants described the ways in which they can deliberately lower their inhibitions, induce temporary antisociality and thereby enable themselves to commit violent and other crimes through strategic drug intake informed by the experience and knowledge obtained from years of carefully monitored substance use.

### Experiencing and understanding uncontrolled substance use and violence during OMT

Participants of the study explain that their moral principles are also compromised by impulsive substance use during OMT. When discussing this matter, they demonstrate keen awareness of the ways in which various substances influence them and their behavior. Frederic had, at the age of 30, served several years in prison due to a pattern of crime, multisubstance use, and violent behavior. He then decided to enroll in OMT to reduce criminal behavior and avoid being imprisoned. However, at the time of the first interview, he had been imprisoned several times during OMT and recalls his most recent prison release. He had been offered a room in an institution but soon broke a house rule and was forced to leave immediately. Fredric despaired:

OK, I'll go all the way [I thought]. I got high - on everything. [..] The police came and I was taken into custody again. I think [this time] I'll get almost a year - violence against a public officer. I kicked his leg and spat on his face. Possession of drugs; resisted arrest violently; was carried to the car. I don't remember it all, I was *so* high. I black out when I take pills.

When asked if certain drugs make him violent, he responds:

It isn't amphetamine, but pills that make me mad and aggressive. Heroin is not my main problem [regarding violence]… But I also have a somewhat aggressive way of being without drugs. It is mainly because of intoxication that I serve sentences. [..] Every time I get arrested for details I resist. That is a problem when I'm high on pills. I feel that I am treated unfairly and resist, and that is what I am sentenced for.

Frederic demonstrates insight into the relationship between his drug intake, hot-tempered character, and violent behavior. He knows well the diverse ways that different drugs affect him and contribute to violence. He has learned that, for him, heroin is protective against violence and amphetamine neutral, and that, in high doses, benzodiazepines increase the risk of becoming violent by reinforcing his otherwise ‘somewhat aggressive way of being’. While he holds intoxication to be instrumental to his violent behavior and subsequent incarcerations, he neither eradicates himself as a subject nor externalizes his violent acts. On the contrary, he draws attention to aggression as a general feature of his mode of being in the world and pattern of reacting violently to unjust ‘details’ when ‘high on pills’. He explains that, in the absence of high-dose benzodiazepine use, he would have a higher tolerance for unjust experiences. As a result of still being imprisoned for violent crimes when enrolled in OMT, Frederic plans to discontinue OMT before being released from prison and instead continue outpatient treatment for benzodiazepine dependence.

While Fredric recalled and, to a certain extent, rationalized what had happened, others have experienced episodes of impulsive and uncontrolled drug use and/or blackouts during which they have violated what they hold to be important moral principles. Afterwards, they have struggled to recall, understand, and reconcile their moral transgressions.

Morten experienced an episode of what he describes as massive and unplanned drug intake, which led to a loss of control and blackout during which he committed a serious violent crime. He had been opioid dependent for nearly two decades and, though he was convicted of several violent and non-violent crimes prior to OMT, claimed to have discontinued all criminal behavior upon OMT enrollment. He had moved out of Oslo to limit his access to illicit drugs and committed himself to avoiding illicit drugs and sedatives in his new hometown to prevent destroying the new life he had built up. For some time prior to imprisonment, he had clean urine tests, worked, and maintained an apartment. However, he experienced increasing social and economic stress and purposively traveled to Oslo to use benzodiazepines on a few occasions. When he felt that he was losing control, he applied for institutional treatment. Meanwhile, he again went to Oslo deliberately to temporarily relieve stress by using benzodiazepines:

I was going to the capital [Oslo] to buy some pills and then [had planned to go] back home again. And I took more and more [pills] plus other things and lost it completely. I don't remember anything, had a real blackout, terrible, and got into deep shit. When I came to my senses I understood nothing, was arrested and in hell. [..] I have spent the entire time of my first two weeks in custody trying to collect myself in order to avoid a complete collapse.

Prior to OMT, Morten had violated his moral standards when financially desperate and, though he managed to accept these transgressions, this was not the case in the situation described above. Rather, Morten found it impossible to understand how he could lose control, relapse completely, and experience a blackout during which he committed a morally off-limits act that he could otherwise never imagine himself capable of. He neither remembered the violent act nor his motive for it, and this seriously threatened his sense of self and led him to the brink of a breakdown.

Ulf, who had previously served several long-lasting sentences for drug, property, and violent crimes, and who organized smuggling of buprenorphine into prison prior to OMT enrollment [36], offers another example of uncontrolled drug use and violence in OMT. While imprisoned, he feels that he can control his drug use. This control, however, was compromised shortly after his most recent release, prior to which he had used central stimulants in prison. He describes his loss of control and substance use as follows:

They wanted to release me to an institution, but I didn't want that. I didn't want to be a part of the treatment system… I wanted to run my own life. Well, I had taken cocaine in prison the last two weeks. When I got out, I went through the last gram of cocaine and a quarter of heroin, plus pills and alcohol. I took it all, plus amphetamine. I went berserk and was taken back to prison.

Going ‘berserk’, in this case, involved committing a severe violent crime, for which he is now imprisoned. Ulf thinks a lot about this episode of uncontrolled and indiscriminate substance intake and, not the least, about the violent crime he committed.

When I think about those days, it eats me, my body twists in disgust. But I know that if I was released now, I would do the same again.

Ulf has undergone several forensic psychiatric evaluations, concluding with an antisocial personality disorder diagnosis. His own understanding of his personality, which he regards as a permanent condition independent of drug use, is based on the years he spent in prison.

I have come to realize that I don't always send good signals … I am immature and have a low development of empathy.

Although Ulf states that he has a low level of empathy, he regrets his violent crime and worries about doing it again if released. Ulf relates the diagnosis to his antisocial behavior, but the diagnosis is not used to disclaim responsibility for his violent behavior and he does not believe that there is a possibility for change. Ulf explains that he can manage life in prison and thinks that he will probably spend most of his adult life incarcerated. So far, by committing high profile crimes, he has, in effect, ensured that his time spent outside of prison is limited. In a way, his unplanned execution of violence might be paradoxically seen as his way of preventing more of the same.

### Moral principles related to violent behavior

As almost all the participants had been imprisoned for violence against others, conceptualizations of violence and crime were among the interview themes. None of the participants regarded violence as external to the law. However, while some regarded the law as *the* undisputable authority in defining crime, others held that situational needs, such as self-defense, motive, or maintenance of subcultural order, should have consequences for whether or not particular acts of violence constitute crime. Erik, for example, made concessions for cases in which he considered physical force to be a rightful and necessary disciplinary reaction *within* drug culture:

You do not cheat, you do not sell bad drugs to the ones you have around you and think of as friends. [..] If someone breaks those rules, then he will be punished - usually. That's the reaction… and he will be beaten up.

Other participants would, as a general principle, recognize violence as a crime but, at the same time, define the crime's severity in accordance with the degree of harm inflicted. For a few, like Frederic, whether or not violence should be regarded as crime depended upon the motive. More concretely, he does not consider self-defense a crime:

I don't feel like a criminal. To me fighting isn't a crime [but] that's what they put me in prison for. Blind violence is a crime. I get pressured and pushed several times. Then I finally hit. To me that is self-defense.

All of the study participants operate according to moral principles, with a few identifying all and the rest identifying certain forms of violence as morally off-limits. Victor provides an example:

Never break into people's homes… Never rob families, old people. I do not rob people on the street. I never beat up people for money, unless they owe me money.

Victor has served many sentences in prison for drug, property, and violent crime. Nevertheless, he explains that, upon each release, he must readjust to life outside of prison and ease back into criminal activity, negotiating and eventually transgressing *his moral principles*.

He has thus developed a routine of beginning with minor criminal acts and gradually habituating himself to more serious, potentially violent, and exhilarating criminal behavior that leads to an excitement rush comparable to the high achieved through substance use. As he explains:

When I come out, I begin carefully. I must do something, because I need to get going again… Yes, you begin to steal a little in stores and you begin to feel the warmth again. You get comfortable with that and eventually begin to think bigger. [..] It is much more exciting to wear a robber's mask and do something than to buy a half kilogram of heroin and stand and sell it. It provides a different type of ‘high’ or kick.

Other study participants try to maintain a high threshold for enacting violent behavior. For example, Paul had been a heroin dealer for many years prior to OMT and had experienced that the demand for heroin declined as OMT became available for a large proportion of opioid-dependent individuals, which made it difficult for him to support himself. When enrolled in OMT, he struggles to avoid being a part of the drug distribution system, but, in certain periods, he still is. He explains why disrespecting certain drug culture norms, such as honoring deals and paying debt, may justify violent consequences:

It takes a lot, at least in my case, for me to use violence and such. But, sometimes, one must do so. One must send signals to others that it is not acceptable. If you do not do so within a certain time frame, more and more will take advantage of you. This is how it is in on the street. If you give a little, they will take everything.

Other times, however, the study participants violate their moral principles without such justifications. The majority of the participants, for example, have experienced that the desperation associated with withdrawal interferes with their ability to uphold all moral principles and increases their vulnerability for committing violent crimes. While heroin dependent, Mona had served several sentences for drug, property, and violent crimes. Some years prior to entering OMT, she made a decision to sell sex [35] as to avoid both violent and non-violent criminal behavior. While enrolled in OMT and using mainly OMT medications, Mona finds it difficult to think about her former life. She describes her experiences and emotions related to many years of heroin use and continuously breaking her own moral codes like this:

When you're high, you lose inhibitions. You have the same moral, I think, somewhere deep inside. And then you get so desperate, mentally - but also physically, especially with heroin…you get so sick and you are so afraid that you'll stay there forever… Then you break your own rules: that you shouldn't steal, that you shouldn't do this and that… even if you know that it's terribly wrong… To justify these actions is one thing, but to understand it is harder. To accept that I was like that and maybe that it's a part of me since I was like that…. That is something I will have to live with the rest of my life. That is something that will never disappear: that feeling of shame and the bad conscience.

## Discussion

Prior to OMT, the participants intentionally induced an ‘antisocial self’ by use of substances - high-dose benzodiazepines, in particular, but also central stimulants and alcohol or combinations of substances. They did so to reduce inhibitions, empathy, and impulse control and, thereby, enable themselves to transgress individual moral codes and perform economically compulsive violent and non-violent crimes, mainly to support costly heroin use. During OMT, impulsive and uncontrolled substance use and subsequent unplanned violent crime was reported. Post-violence use of benzodiazepines was reported to reduce memories of and alleviate the emotional unease and guilt associated with this violence.

The participants operate according to moral codes that often parallel logics of morality commonly found throughout both mainstream and drug cultures that serve important functions within drug culture and that designate the conditions under which violent crime is and is not justified and the types of violence that are and are not permissible. While some participants rationalize their violent behavior, others struggle to reconcile the resulting shame, guilt, and fractured sense of self. This study's findings suggest that a violation of one's moral principles cannot be read as a lack of moral principles entirely; on the contrary, in order to break a rule, the rule needs to exist in the first place.

Shame and guilt are ‘self-reflective’ moral emotions [44]. Rather than using cognitive neutralization techniques described by other researchers, such as using self-talk to deny responsibility, injury, and the victim, condemning the condemners, and appealing to higher loyalties to reduce feelings of guilt *before* they break the law [45-47], the participants in this study report substance use and use of high-dose benzodiazepines, in particular, prior to committing (violent) crimes to achieve the same effect. Use of ‘unusually high doses of benzodiazepines’ was found to be related to high violence risk, and a low dose was found to reduce the violence risk in a sample of remand prisoners where the most commonly reported motivation for taking benzodiazepines were ‘reduced anxiety’ and ‘feeling better [23]. Benzodiazepines are also known to affect memory [48-50], and use of flunitrazepam is reported to lead to anterograde amnesia [51]. In this study, we find that the study participants deliberately use these substances to reduce memory and feelings of guilt *after* having committed a violent crime.

A study among imprisoned violent offenders found research interviews of importance for the participants to ‘construct themselves as morally decent persons’ [52]. This was also of importance for our study participants. But, for these study participants, to morally justify violent and non-violent behavior seemed to be easier while using heroin prior to OMT, as some individuals found it difficult to be enrolled in OMT and no longer able to use substances to reduce thinking and emotions related to previous criminal behavior. We suggest that some individuals might experience simultaneous OMT enrollment and imprisonment for a (violent) crime to elicit feelings of ‘double’ shame and guilt for both their criminal behavior prior to treatment and the actual case(s) they are imprisoned for while in treatment.

Some study limitations should be recognized. The participant sample was highly selective and the findings cannot be generalized in a statistical sense, but should instead be considered for the ways that they contribute to a more nuanced understanding of some OMT-enrolled individuals' experienced relationship between substance use and violent crime and the potential clinical implications. The interviews took place in prison, were conducted by a mental health practitioner, and addressed sensitive topics—all of which may have influenced the participants' retrospective reflections, their decisions to share particular experiences, and their understandings of these events and their consequences [53], possibly encouraging them to provide a positive self-presentation, as seen from their perspective.

## Conclusions

In a clinical OMT setting, treatment providers may take measures to identify individuals with histories of violent behavior while under the influence of substances, intoxicated, or in a state of blackout. These individuals may be capable of reflecting upon and problematizing their violent behavior in treatment. Violent behavior is situational, and the concrete situations that precede, motivate, and contextualize violence, as well as the potential role of substances, should be matters of joint exploration between treatment providers and OMT patients to tailor individual treatment approaches accordingly. Some individuals may be at risk for further violent behavior if they continue to use substances while in OMT, and treatment for poly-substance dependence and, in particular, benzodiazepine dependence, may be provided. Furthermore, feelings of remorse, guilt, and shame related to violent and non-violent crimes both prior to and during OMT are of importance throughout treatment and especially among (remand) prisoners. When assessing personality disorders in OMT populations, the identification of dysfunctional traits should demand stability over time and consistency across situations.

## Abbreviations

OMT: opioid maintenance treatment.

## Competing interests

The authors declare that they have no competing interests.

## Authors’ contributions

TC was the project manager. TC, IH, and ALM conceived the qualitative project and constructed the interview guide. IH conducted the interviews. IH, CB, and ALM conducted the analysis and wrote up the first draft of the paper. All authors contributed to and have approved the final manuscript.
